# Regulatory Mechanisms and Functional Roles of Readthrough Transcripts in Tumorigenesis

**DOI:** 10.3390/ijms26209975

**Published:** 2025-10-14

**Authors:** Alexander Modestov, Galina Zakharova, Elena Poddubskaya, Anton Buzdin

**Affiliations:** 1 Institute for Personalized Oncology, Biomedical Science & Technology Park, FSAEI HE I.M. Sechenov First Moscow State Medical University of MOH of Russia (Sechenovskiy University), 119991 Moscow, Russia; modestov_a_a@staff.sechenov.ru (A.M.);; 2 Burnasyan Federal Medical Biophysical Center, Federal Medical-Biological Agency of Russia, 123098 Moscow, Russia; 3 Scientific Center of Genetics and Life Sciences, Sirius University of Science and Technology, 354340 Sirius, Russia

**Keywords:** downstream-of-gene transcripts, DoG, cis-splicing of adjacent gene transcripts, cis-SAGe, gene readthrough, fusion cancer transcripts, RNA sequencing, NGS, cancer progression, patient prognosis and survival biomarkers

## Abstract

The search for novel tumor-specific markers and therapeutic targets is driving the development of more effective and personalized treatment strategies for cancer patients. This article focuses on investigating a promising new source of biomarkers—readthrough transcripts, or downstream-of-gene (DoG) transcripts. These transcripts are extended products of gene transcription that continue into intergenic regions and can overlap neighboring genes, sometimes giving rise to cis-splicing of adjacent gene (cis-SAGe) transcripts. Recent studies suggest that besides frequently being a “transcriptional noise”, DoG transcripts can perform regulatory functions, serve as a source of novel protein products, and act as prognostic markers of patient survival across various cancers. This article aims to investigate the regulatory mechanisms and functional significance of readthrough transcripts in tumors, to identify currently known tumor-specific variants with potential utility as cancer biomarkers or therapeutic targets, and to evaluate the most suitable approaches for their detection. The knowledge gained through this research may provide a foundation for the development of diagnostic test systems and the design of new anticancer drugs.

## 1. Introduction

**Figure 1:**
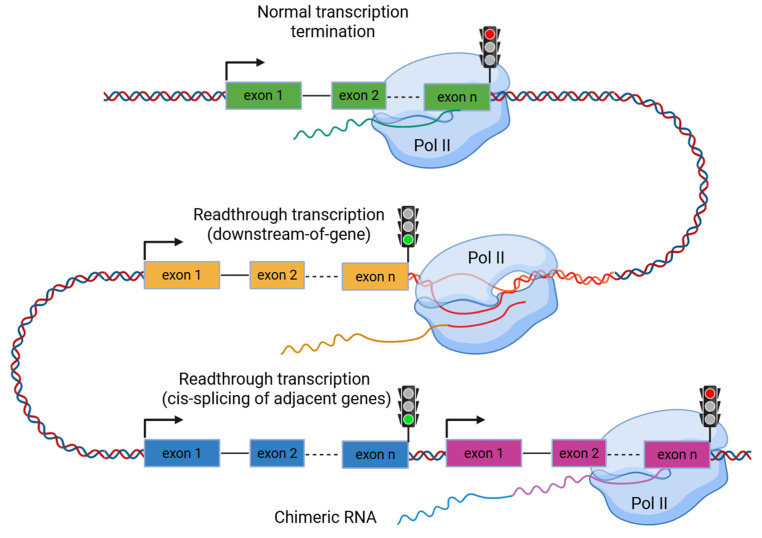
Readthrough transcription events. Failure of transcription termination and readthrough transcription can lead to read-in transcription of a downstream gene by RNA polymerase II (Pol II). The phenomenon of readthrough transcription can generate two main types of transcripts: those that fail to terminate at the canonical 3′-end, and those that extend into a neighboring gene, resulting in cis-splicing of adjacent gene transcripts.

In the human genome, the number of protein-coding genes is markedly lower than the number of transcripts, owing to mechanisms, such as RNA editing, alternative splicing, alternative transcriptional start and termination, and other post-transcriptional modifications. One critical step in this regulation is transcription termination by RNA polymerase II, a tightly controlled process involving the cleavage of nascent RNA and the release of the polymerase to ensure precise production of mature mRNAs. As RNA polymerase II reaches the 3′-end of a gene, it transitions into a termination phase orchestrated by RNA cleavage, polyadenylation, and the subsequent disassembly of the transcriptional complex (Figure 1). Under certain conditions, the transcription machinery may fail to recognize the termination site and continue transcription beyond the annotated gene boundaries, a phenomenon known as readthrough transcription, or downstream-of-gene (DoG) transcription (Figure 1).

Initially, it was found that activation of DoG transcript production in cells occurs in response to stress stimuli, such as osmotic [1], heat shock, or oxidative stress [2]. Notably, this stress-induced readthrough is not random; rather, its activation pattern depends on the type of stress applied [2]. Later, it was revealed that this phenomenon may be more widespread than merely a stress response. For instance, readthrough transcription also occurs in healthy tissues, albeit apparently at lower levels [3]. Under normal conditions, DoG transcription varies with tissue type and age of a subject, showing increased levels in tissues obtained from aged donors and in senescent cells. Elevated DoG formation is also detected in cortical brain tissue during Alzheimer’s disease [4]. Although in the context of aging the specificity of DoG expression has been questioned, it has been proposed that this phenomenon may simply reflect transcriptional noise associated with aging [5]. However, in this review of primary interest is the study of readthrough transcription in the context of tumorigenesis. A recent study revealed tumor type- and stage-specific features of DoG transcript expression [6]. Moreover, suppression or activation of specific DoG transcripts has been linked to poor survival prognosis in breast, colorectal, liver, and kidney cancers [6,7].

In certain cases, readthrough transcription can produce chimeric transcripts known as cis-splicing of adjacent gene (cis-SAGe) transcripts (Figure 1). This phenomenon arises when two genes oriented in the same direction on a chromosome are transcribed together as a single unit. In such events, cis-SAGes arise through intergenic splicing of a precursor RNA that spans both neighboring genes. Normally, transcription terminates at well-defined regulatory signals, preventing RNA polymerase II from extending into the downstream gene, and the intergenic region separating adjacent genes is generally excluded from pre-mRNA synthesis. However, during cis-SAGe formation, termination signals are bypassed, the intergenic region is transcribed into the pre-mRNA, and subsequently removed as if it were an intron through intergenic splicing. As shown in recent studies, these events constitute a major part of events leading to formation of chimeric transcripts in colorectal [8] and neuroendocrine [9] tumors, but they are also frequent in normal tissues like pituitary gland [9].

The essential requirements for cis-SAGe formation include active transcription of the upstream gene and escape from normal transcriptional boundaries. Additionally, it requires the generation of a single pre-mRNA containing sequences from both genes and the intervening intergenic region, as well as the production of a mature spliced mRNA incorporating exons from both genes. Dysregulation of the molecular processes governing these steps can directly affect the synthesis of cis-SAGes. Similarly to chimeric genes formed as a result of chromosomal rearrangements, such transcripts can encode novel proteins, some of which may possess oncogenic properties and, therefore, could be used as biomarkers or even targets for anticancer therapy.

Readthrough transcripts represent a promising niche for studying the mechanisms of gene regulation in tumors, stress responses, survival pathways in response to therapy, and for the discovery of novel biomarkers and therapeutic targets. This article comprehensively investigates published reports of the phenomenon of readthrough transcription, ranging from the identification of tumor-specific DoG expression patterns to the elucidation of the mechanisms regulating them and their potential functional roles.

## 2. Biological Mechanism of Readthrough Transcription

Initially, the phenomenon of readthrough transcription was studied in the context of cellular responses to stress stimuli [1,2]. The resulting DoG profiles differed depending on the type of stress [2]. Later, it was found that DoG transcript production also occurs in normal tissues [3,4]. Similarly, the nature of readthrough transcript formation was shown to depend on both tissue type and age [4]. In tumors, specific patterns of readthrough transcript expression have been observed, characteristic of tumor type and stage. Moreover, DoG expression patterns can be used to predict patient survival in breast, colorectal, liver, and kidney cancers [6,7].

**Figure 2:**
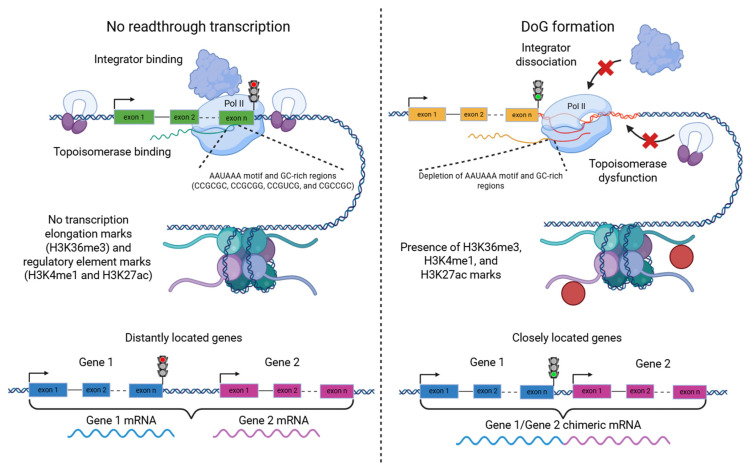
Factors influencing the formation of readthrough transcripts. It may be associated with Integrator complex dysfunction, topoisomerase I dysfunction, depletion of polyadenylation motifs and GC-rich regions, chromatin accessibility, and the distance between genes. Pol II—RNA polymerase II.

It is known that DoGs are synthesized by RNA polymerase II [10,11] and represent an extension of the mRNA of their associated genes. Their length can range from 2 kb to over 100 kb, although they most commonly occur within the 5–10 kb range [2,4,12]. DoG expression correlates with the transcriptional activity of the associated driver 5′-gene [4,12], meaning that high DoG production requires high expression of the initiating gene, while suppression of that gene’s expression leads to decreased DoG production [12]. However, high gene expression alone is not sufficient for DoG formation [12], indicating that additional factors are required [10]. The main factors influencing the formation of both readthrough transcripts, in general, and cis-SAGe transcripts in particular can be grouped into the following categories (some of them are presented in Figure 2):

Transcription termination

(A) Integrator complex dysfunction. Normally, the Integrator complex binds to a stalled RNA polymerase II elongation complex and induces transcription termination [13]. Stress-induced activation of DoG transcripts is associated with dissociation of the Integrator complex from RNA polymerase II, with the INT11 subunit playing a key role in this process [10,11].

(B) Topoisomerase I dysfunction. Recent data show that topoisomerase 1 (TOP1) is involved not only in transcription initiation and elongation but also plays an important role in proper termination. Suppression of TOP1 leads to more frequent DoG activation, while its overexpression results in reduced DoG transcript production [6].

Pre-mRNA processing

(A) Defects in cleavage and polyadenylation of nascent pre-mRNA. It is known that suppression of the catalytic subunit of this complex, the endonuclease CPSF73, leads to increased formation of readthrough transcripts [12,14].

(B) Pre-mRNA splicing defects. Several splicing-related factors have been shown to influence cis-SAGe transcript formation. For instance, SRRM1 suppression decreases the production of chimeric transcripts, while SF3B1 suppression increases it [15]. However, splicing defects can also affect the formation of readthrough transcripts more broadly. For instance, suppression of U2AF1 leads to increased DoG transcript formation [4]. Inactivation of both SF3B1 and U2AF1 also results in other splicing abnormalities, such as exon skipping, intron retention, and others [15,16]. In certain normal tissues, such as liver, skeletal muscle, and esophageal mucosa, the presence of DoG transcripts has been associated with an increased frequency of intron retention in the corresponding gene, particularly in the last two introns [4]. The link between readthrough transcription and splicing is further supported by the observation that intronless genes rarely produce readthrough transcripts, with fewer than 5% of such genes doing so. Additionally, the likelihood of DoG formation increases with the number of introns in a gene, reaching a plateau of about 30% for genes containing 20 introns [4].

Genomic context

Accumulating evidence indicates that the three-dimensional organization of chromatin influences not only transcription initiation and elongation but also plays a critical role in transcription termination. Alterations in chromatin architecture can modulate RNA polymerase II dynamics, thereby affecting both the efficiency and fidelity of the termination process.

(A) Depletion of polyadenylation motifs. In the study of stress-activated DoG production, it was shown that such transcripts are more likely to arise from genes in which the transcription termination site region and the downstream sequence are depleted of canonical polyadenylation motifs (AAUAAA) [2]. In another study on normal tissues, these findings were not confirmed; however, it was found that for genes producing DoGs, the ~500 nt regions upstream and downstream of the transcription termination site exhibit reduced frequencies of GC-rich oligomers, such as CCGCGC, CCGCGG, CCGUCG, and CGCCGC. It has been proposed that such GC-rich sequences may slow down the elongation complex, thereby providing more time for the cleavage and polyadenylation complex to locate the polyadenylation site [4].

(B) Disruption of insulator function. Several studies associate the formation of readthrough transcripts with the involvement of the CTCF factor [17], specifically with reduced CTCF binding at corresponding intergenic regions. It is noteworthy that CTCF can also promote the formation of chimeric RNAs by bringing distant genes into close spatial proximity, thereby facilitating the generation of trans-splicing chimeric RNAs. An example of such a chimera is *JAZF1–JJAZ1*, whose expression was reduced upon CTCF silencing [18], indicating that CTCF may exert differential effects on chimeric RNA formation depending on the underlying mechanism. Notably, cis-SAGe events could be independent of CTCF regulation [17].

(C) Chromatin accessibility. Genes that generate readthrough transcripts show increased enrichment of transcription elongation marks (H3K36me3) and regulatory element marks (H3K4me1 and H3K27ac) at the 3′-end region of the gene and downstream of the polyadenylation site. These genes also frequently exhibit DNase I hypersensitivity sites downstream of the polyadenylation site. This is observed not only under stress conditions but also in normal states, suggesting that an open chromatin conformation facilitates DoG formation [2,4]. However, mutations or suppression of the histone methyltransferase SETD2, which is responsible for H3 lysine 36 methylation, lead to increased DoG production despite the presence of H3K36me3 [7]. This indicates that not merely the presence, but also the distribution pattern of H3K36me3 is important. Based on histone mark distribution, it has also been suggested that active enhancers may serve as barriers to readthrough transcription [4].

(D) Immediate gene neighborhood. Interestingly, DoGs are more frequently formed when another gene is located near the 3′-end of the primary gene, regardless of its orientation [2].

If the formation of DoG transcripts is not the result of random regulatory failure but instead displays a differential pattern depending on the type of stimulus [2], then it is reasonable to expect that these transcripts may have specific functions.

Stress-induced DoGs in the cell are predominantly co-localized with chromatin [12]. This may indicate that a possible role of DoGs in the stress response is to support the functional organization of chromatin, likely through stabilizing interactions between chromatin and the nuclear matrix. However, in the case of herpes simplex virus type 1 (HSV-1) infection, DoG transcripts remain not only chromatin-associated but are also distributed throughout the nucleoplasm [19]. Additional evidence supporting the hypothesis of a role in chromatin organization comes from other studies. For example, the presence of readthrough transcription has been shown to alter chromatin accessibility downstream of the gene’s polyadenylation site. During influenza A virus infection, transcription termination was disrupted by the viral NS1 protein, leading to the formation of readthrough transcripts. Elongation of RNA polymerase II beyond the gene boundaries, in turn, caused chromatin decompaction through displacement of cohesin from CTCF-binding sites [20]. During HSV-1 infection, a similar pattern emerged, characterized by the induction of readthrough transcription and increased chromatin accessibility in downstream genomic regions [19]. Notably, given the earlier observation that open chromatin is required for readthrough transcript production, it remains unclear which event occurs first. The link between chromatin accessibility and DoG production is likely to involve more complex regulatory mechanisms, warranting further investigation. DoGs and long non-coding RNAs may also act as regulatory sponges adsorbing miRNAs [4,21]. Additionally, they may participate in maintaining chromatin structure [20] and influence the expression of neighboring genes either through antisense effect [2] or via competitive mechanisms [22].

Cis-SAGe transcripts

Another important case of readthrough transcription is the formation of chimeric mRNAs through cis-splicing of a shared transcript from two co-oriented genes, known as cis-SAGe. Between 10 and 20% of all genes in healthy tissues produce such transcripts, predominantly protein-coding genes [4]. Chimeric cis-SAGe transcripts can constitute up to 34% of active protein-coding genes under normal conditions [4] and up to 55% under stress [10]. Most chimeric transcripts are formed through splicing at the canonical sites of both participating genes [3]. Typically, the junction connects the penultimate exon of the upstream (5′) gene with the second exon of the downstream (3′) gene [17,23]. Similarly to DoGs, cis-SAGe chimeric transcripts show expression patterns that correlate with the 5′-gene [24], but not with the 3′-gene.

**Figure 3:**
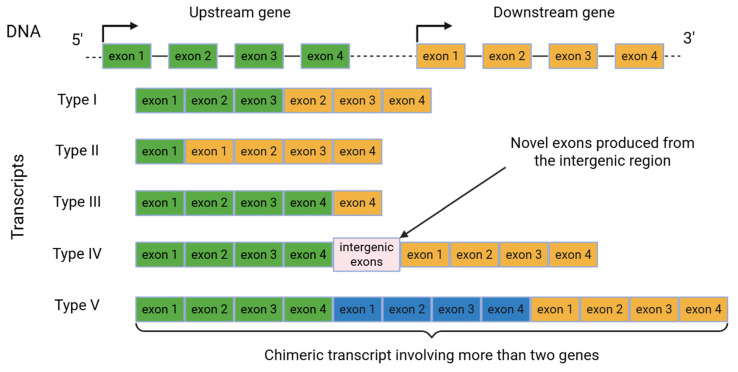
Five types of cis-SAGe transcripts illustrated using genes containing four exons.

Several classifications of cis-SAGe have been proposed depending on the exon composition of the participating genes [25,26]. For instance, the study [25] describes five major types (Figure 3). Type I, the most frequently observed, occurs when transcription bypasses the termination site located at the 3′-UTR of the upstream gene, extending into the downstream gene while excluding the last exon of the upstream gene and the first exon of the downstream gene. Type II links the first exon of the upstream gene, which may have regulatory motifs for proper transcription regulation, with exons of the downstream gene. Type III involves exons of the upstream gene and the last exon of the downstream gene and contributes to transcription termination efficiency. Type IV incorporates novel exons from the intergenic region, whereas Type V involves more than two genes in chimeric transcript formation.

In a study of virus-induced DoG expression, it was hypothesized that readthrough transcripts remain predominantly in the nucleus because they cannot be efficiently exported [19]. This effect may partly be related to the infection itself. It may also be explained by the fact that aberrant splicing and retained introns can cause cis-SAGe transcripts to be sequestered in the nucleus, thereby preventing translation of aberrant mRNAs [22]. However, this mechanism does not apply to all cis-SAGe transcripts. Several studies have shown that chimeric cis-SAGe transcripts can be translated. For example, in nontumor tissues and cell lines, two chimeric transcripts, *CTBS-GNG5* and *CTNNBIP1-CLSTN1*, were found to produce protein products, and silencing these chimeras led to reduced cell growth and motility [3]. Notably, the study [1] demonstrated that cis-SAGes and their corresponding DoGs exhibit an inverse correlation during the initial phase of osmotic stress, when the total amount of readthrough transcripts is limited. This finding supports the idea of a competition between DoG transcription and chimeric RNA formation. At the later phase of osmotic stress (24 h), however, this negative correlation shifts to a positive one, as both DoGs and cis-SAGe RNAs become induced. Yet, the precise link between DoGs and cis-SAGes remains unclear, and the functional interplay between these two distinct phenomena requires further investigation.

Readthrough circular transcripts (rt-circRNAs)

A special subtype of cis-SAGe transcripts is represented by readthrough circular RNAs, or rt-circRNAs [27,28]. These are chimeric transcripts that contain exons from two adjacent genes but are additionally covalently closed into a circular structure. Circularization during backsplicing is facilitated by complementary pairing between intronic regions enriched in repetitive sequences. The rt-circRNAs typically comprise downstream exons of the 5′-gene and upstream exons of the 3′-gene. Compared with other circRNAs, rt-circRNAs have longer introns with abundant repetitive elements [28]. rt-circRNAs constitute only a small fraction of all circRNAs (2.5% per sample), with a total of 1359 rt-circRNAs identified. Among them, *AMACR-SLC45A2* and two *KLK3-KLK2* isoforms are prostate-specific, whereas *C4A-C4B*, *ORM1-ORM2*, *SDC2-CPQ*, *CYP2C18-CYP2C19*, and two isoforms of *HP-HPR* and *ADH1B-ADH1A* are liver-specific [27]. Notably, among these rt-circRNAs, only *AMACR-SLC45A2* and *SDC2-CPQ* are derived from non-paralogous genes. Moreover, *AMACR-SLC45A2* and both *KLK3-KLK2* isoforms were not detected in normal tissues. In total, 817 rt-circRNAs were absent from normal tissues, suggesting their potential involvement in tumor development.

In the study on liver cancer, researchers identified a *CYP2C18-CYP2C19* readthrough circular RNA, termed rtcisE2F, which is associated with tumor development and is highly expressed in tumor tissues [29]. It was found that rtcisE2F acts as a scaffold to promote the interaction between IGF2BP2 and *E2F6/E2F3* mRNAs, thereby maintaining their stability and driving the self-renewal of liver cancer cells. In addition, rtcisE2F inhibits YTHDF2 binding to *E2F6/E2F3* mRNAs, which further decreases their degradation. Targeting components of the rtcisE2F–IGF2BP2/YTHDF2–E2F6/E2F3 axis may therefore represent a potential strategy for liver cancer therapy.

Little is known about this group of readthrough transcripts. Further studies are needed to elucidate their possible functions as miRNA sponges, contributions to tumorigenesis, and potential as biomarkers and therapeutic targets.

## 3. Functional Roles of Readthrough Transcription

**Figure 4:**
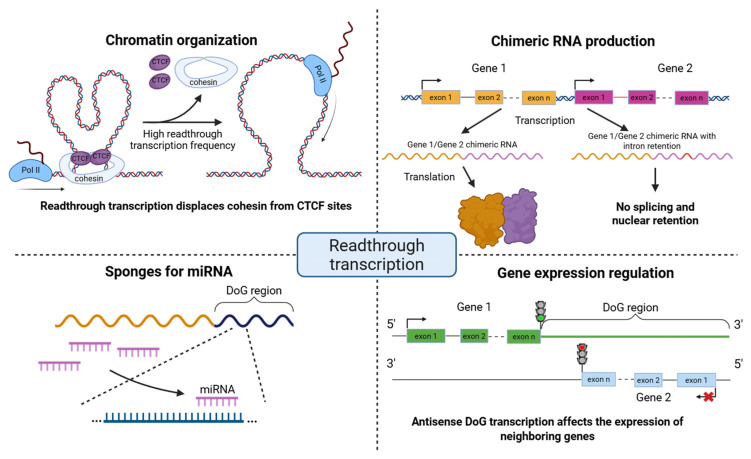
Schematic representation of the functional role of readthrough transcripts. Readthrough transcription can affect the production of RNA chimeras, chromatin organization, miRNA regulation, and expression of neighbor genes. Pol II—RNA polymerase II, CTCF—CCCTC-binding factor.

DoGs are increasingly recognized as a hallmark of transcription termination defects [10,12,20,30]. Their biological roles are diverse, affecting multiple cellular processes, including the production of RNA chimeras, chromatin organization, miRNA regulation, and the expression of neighboring genes [2,4,20,22] (Figure 4). In particular, there are several studies providing evidence that readthrough transcription can exert profound effects on gene expression [2,22]: readthrough transcripts can extend into the neighboring genes, thereby repressing their activity through transcriptional interference, or give rise to RNA chimeras. Chimeric transcripts spanning multiple genes with retained introns have been observed when splicing fails due to disruption of NAB2, a factor essential for proper 3′-end cleavage [31,32]. Beyond transcriptional interference, readthrough transcription has also been implicated in shaping host genome 3D organization [20]. In that study, the viral non-structural protein NS1 promotes readthrough transcription of highly active genes, leading to cohesin displacement from chromatin, loss of chromatin loops, and decompaction of chromatin within the readthrough regions. Further studies will be necessary to disentangle the relative contributions of NS1-mediated disruption of mRNA processing, widespread readthrough transcription, and altered chromatin structure in the suppression of host antiviral responses and the enhancement of viral virulence. In addition, DoGs have been detected within the chromatin-bound RNA fraction, where they appear to colocalize with their upstream DoG-producing transcripts [12]. Given their considerable length, this observation suggests a potential role for DoGs in reinforcing the nuclear scaffold. Further investigation will be required to determine whether DoGs influence gene expression directly, in addition to contributing to nuclear integrity and stabilizing genomic regions that promote their own formation. Therefore, it is important to analyze the hallmarks of readthrough transcription that give rise to DoGs and cis-SAGes in healthy and cancer tissues.

In the study [4], transcriptional readthrough was examined in 2778 transcriptome profiles from 23 human tissues, revealing that about one third of expressed protein-coding genes produced readthrough transcripts. Their occurrence was limited in regions of high transcriptional activity and correlated with inefficient splicing and increased chromatin accessibility at terminal regions. Moreover, multiple miRNA-binding sites have been identified in these transcripts, which points to their possible function as miRNA sponges. Collectively, these findings indicate that readthrough transcription is widespread and non-random, occurring not only under pathological conditions but also in healthy tissues, where it may contribute to cellular homeostasis.

Further complex analysis confirmed readthrough across diverse healthy tissues, with at least 30% of all protein-coding genes producing readthrough transcripts [4]. Of the 7138 readthrough genes identified across all tissues, ~85% were protein-coding genes. Interestingly, approximately two thirds of readthrough transcripts were detected in at least two tissues. Enrichment of histone marks associated with transcription elongation H3K36me3 was detected at the 3′ ends of readthrough genes, spreading past the termination boundaries. In some tissues, additional enrichment of regulatory chromatin marks H3K4me1 and H3K27ac was detected downstream of termination sites, indicating that these chromatin features are also associated with readthrough transcription under normal physiological conditions.

Since reduced co-transcriptional splicing efficiency can give rise to termination defects [33], it is also important to investigate the link between inefficient splicing and transcriptional readthrough in healthy tissues. In multiple tissues, including liver and skeletal muscle, readthrough genes exhibited greater levels of intron retention than non-readthrough genes [4]. Inhibition of U2AF1, a key splicing factor, was also found to be associated with an increased number of readthrough transcripts and widespread intron retention [16].

In addition, 1576 readthrough genes (~20% of all readthrough genes) as putative sponges for 2276 miRNAs (~85% of all analyzed miRNAs) were found, and 13 of the 145 miRNAs, for which sponges were confined to one or two tissues, showed elevated expression in the corresponding tissues [4]. Also, less than 1% of readthrough transcripts across tissues were found to extend into adjacent downstream genes, many of which corresponded to long non-coding RNAs.

In the complex study [34], readthrough events were systematically collected and analyzed across 43 healthy human tissues, yielding 75,248 events derived from 35,720 transcripts corresponding to 11,692 genes. The resulting dataset includes sequence information, genomic locations, expression profiles, and detailed gene annotations for readthrough transcripts. This resource provides a comprehensive overview of readthrough transcriptomics and its regulatory significance, serving as a valuable benchmark for future studies.

In the study [6], among the molecular features of DoG RNAs, tumors exhibited alterations in both the number of DoGs and the strength of their extension compared to paired normal tissues. Specifically, moderate increases in DoG number and significantly longer RNA extensions were observed in colorectal cancer and liver cancer tumors, whereas breast cancer tumors displayed a decreased DoG number with non-significant changes in extension strength. Consistent with the cancer-related relevance of aberrant DoG transcripts, the ratio of correctly transcribed mRNAs to those with DoG extensions was reduced in colorectal and liver tumors relative to paired normal tissues. In contrast, breast cancer samples exhibited an increased proportion of properly terminated mRNAs compared to DoG transcripts, implying that alterations in DoG RNA levels may influence tumorigenesis in a tissue-specific manner [6]. Recent studies examining gene-length shifts provide a framework for age-associated imbalances in transcript length [35], although the causal roles of length-associated transcripts, including DoG RNAs, in normal development and disease remain to be determined.

Cancer progression involves multiple genetic and epigenetic alterations, encompassing both gain-of-function oncogenes and loss-of-function tumor suppressors. Comparative analysis of DoG RNA signatures across paired normal tissues and tumors of breast, colorectal, and liver origin revealed both up- and down-regulation of DoG expression [6]. Up-regulated DoG-producing genes were enriched for tumor-promoting pathways, including G2/M checkpoint, glycolysis, and E2F targets, whereas genes with down-regulated DoG RNAs were associated with normal cellular processes, developmental pathways, and tumor-suppressor functions. Consistently, both the up- and down-regulation of DoG RNAs significantly correlated with poor patient survival, indicating that dysregulation of DoG RNA expression aligns with the biological hallmarks of tumorigenesis and tumor suppression.

## 4. Readthrough Transcription Events in Cancer Progression

In routine analysis of transcriptome data, anything beyond the boundaries of annotated genes is usually left out of the researcher’s focus. Typically, DoG transcripts are simply not accounted for in gene expression analysis, and cis-SAGe transcripts are usually filtered out as possible artifacts in the search for chimeric genes using RNA sequencing data. This is primarily due to the fact that tools for analyzing readthrough transcripts have not yet been incorporated into protocols for analyzing transcriptome profiles; moreover, sufficient information has not yet been accumulated to interpret the results obtained. Nevertheless, recent studies show that readthrough transcripts have great potential as biomarkers of tumorigenesis. It has been shown that DoG expression profiles can be specific for tumor type and stage and can also act as prognostic markers of survival, for example, in breast, colorectal, liver, and kidney cancers [6,7]. In particular, readthrough transcripts, especially cis-SAGes, can be a source of novel proteins and neoantigens (Table 1).

In the study [36], expression of long non-coding RNA (lncRNA) *ZFPM2-AS1* was found to be significantly elevated in hepatocellular carcinoma tissues compared to adjacent normal tissues, and higher *ZFPM2-AS1* levels were strongly associated with poor patient survival. Silencing of lncRNA *ZFPM2-AS1* suppressed cell proliferation, migration, and invasion while promoting apoptosis in vitro. Analyses using the lncRNA–miRNA–mRNA interaction databases (miRcode and TargetScan) indicated that lncRNA *ZFPM2-AS1* regulates *GDF10* expression by competitively binding to miR-139. This study also demonstrated that miR-139 and down-regulation of *GDF10* reversed the cellular phenotypes induced by *ZFPM2-AS1*.

In tumors, numerous examples have been identified in which chimeric cis-SAGe transcripts exhibit oncogenic properties and can serve as biomarkers or therapeutic targets. These include *SLC45A3-ELK4*, *MSMB-NCOA4*, *D2HGDH-GAL3ST2*, and *DUS4L-BCAP29* in prostate cancer [18,24,37,38,39], *SCNN1A-TNFRSF1A* and *CTSD-IFITM10* in breast cancer [40], *LHX6-NDUFA8* and *SLC2A11-MIF* in cervical cancer [41], *BCL2L2-PABPN1*, *HNRNPA1L2-SUGT1* and *CHFR-GOLGA3* in bladder cancer [42,43], *RRM2-C2orf48*, *METTL21B-TSFM*, *SF3A2-AMH*, and *TMEM189-UBE2V1* in colorectal cancer [44,45], *CTSC-RAB38*, *KDSR-BCL2*, and *KLK4-KRSP1* in kidney cancer [7,46], *BCL2L2-PABPN1* in glioblastoma [47], etc. (Table 1). In addition, 16 cis-SAGe candidates were identified by silencing transcription factor CTCF with small interfering RNA (siRNA) in the prostate cancer study [17]. Intergenic transcripts were then detected by reverse transcription polymerase chain reaction (RT-PCR) to further validate these cis-SAGe candidates.

**Table 1:** Experimentally validated cis-SAGes participating in cancer progression.

cis-SAGes (Upstream Gene–Downstream Gene)	Tumor Type	Reference
*SLC45A3-ELK4*	prostate cancer	[18,24,37,48,49]
*BCL2L2-PABPN1*	bladder cancer, glioblastoma	[42,47]
*RRM2-C2orf48*	colorectal cancer	[44]
*METTL21B-TSFM*	colorectal cancer	[44]
*SF3A2-AMH*	colorectal cancer	[44]
*TMEM189-UBE2V1*	colorectal cancer	[45]
*POLA2-CDC42EP2*	gastrointestinal stromal tumors	[50]
*C8orf42-FBXO25*	gastrointestinal stromal tumors	[50]
*STX16-NPEPL1*	gastrointestinal stromal tumors	[50]
*RPL17-C18orf32*	gastric cancer	[51]
*PRR5-ARHGAP8*	gastric cancer	[51]
*PHOSPHO2-KLHL23*	gastric cancer	[51]
*MSMB-NCOA4*	prostate cancer	[24]
*D2HGDH-GAL3ST2*	prostate cancer	[38]
*SCNN1A-TNFRSF1A*	breast cancer	[40]
*CTSD-IFITM10*	breast cancer	[40]
*LHX6-NDUFA8*	cervical cancer	[41]
*SLC2A11-MIF*	cervical cancer	[41]
*TWE-PRIL*	T- and B-lymphoma	[52]
*HNRNPA1L2-SUGT1*	bladder cancer	[43]
*CHFR-GOLGA3*	bladder cancer	[42]
*CTSC-RAB38*	kidney cancer	[7]
*KDSR-BCL2*	kidney cancer	[7]
*KLK4-KRSP1*	kidney cancer	[46]
*TSNAX-DISC1*	endometrial cancer	[53]
*INS-IGF2*	non-small-cell lung cancer	[54]
*DUS4L-BCAP29*	prostate cancer, gastric cancer	[39]
*JMJD7-PLA2G4B*	human head and neck squamous cell carcinoma	[55]
*NFATC3-PLA2G15*	T-acute lymphoblastic leukemia	[56]

Recent studies suggest that cis-SAGes may play functional roles in cancer and should therefore be considered in readthrough detection analyses. A well-characterized example is the cis-SAGe between *SLC45A3* and the ETS transcription factor *ELK4* in prostate cancer [18,24,37,48,49]. *SLC45A3-ELK4* identified via RT-PCR occurs at a low level relative to wild-type *ELK4* and acts as a lncRNA modulating cancer progression. Silencing of *SLC45A3-ELK4* was found to inhibit the proliferation of prostate cancer cells [49].

Three highly expressed, colorectal cancer-biased chimeric RNAs, *RRM2-C2orf48*, *METTL21B-TSFM*, and *SF3A2-AMH*, were validated by RT-PCR and Sanger sequencing [44]. Expression of *RRM2-C2orf48* was associated with poor clinical outcomes, whereas expression of the parental genes *RRM2* and *C2orf48* correlated with favorable outcomes. Functional assays demonstrated that silencing *RRM2-C2orf48* reduced proliferation of colon cancer cells, while overexpression of the chimera enhanced proliferation. Recurrent chimeric RNAs are present in colorectal cancer and may exhibit distinct expression patterns and functions compared to their parental genes, thereby constituting a novel repertoire of biomarkers and therapeutic targets [44].

Another previous study reported elevated expression of the cis-SAGes *BCL2L2-PABPN1* and *CHFR-GOLGA3*, predominantly localized in the nucleus, in bladder cancer [42]. In addition, the cis-SAGe chimera *HNRNPA1L2-SUGT1* was also identified in a separate investigation of bladder cancer [43].

In another study [46], *KLK4-KRSP1* was detected in ~30% of renal cell carcinomas, while being rarely expressed in normal tissue. *KLK4-KRSP1* expression correlated with worse clinical outcomes in the analyzed patient cohort. Additionally, readthrough events influenced molecular mechanisms such as target gene expression and cell migration, opposing the *KLK4* transcript.

In the study [51], readthrough transcription events were screened from stomach adenocarcinoma RNA-seq data, leading to the identification of three candidates, *PHOSPHO2-KLHL23*, *RPL17-C18orf32*, and *PRR5-ARHGAP8*, for evaluation of their biological relevance in gastric cancer.

The cis-SAGe *LHX6-NDUFA8* was determined in cervical cancer cells, with no detectable expression in normal cells [41]. This investigation also brought attention to another recurrent cis-SAGe absent in normal tissues, *SLC2A11-MIF*, whose silencing induced cell cycle arrest and decreased cellular proliferation. Notably, this effect was specific to the readthrough transcript and was not observed for the individual parental genes [41].

While it is important to identify promising tumor-specific readthrough transcripts with potential clinical applications, it is also necessary to investigate them from the perspective of tumor therapy. For example, it is known that inhibition of topoisomerase 1 (TOP1) leads to an increase in the formation of readthrough transcripts, while TOP1 inhibitors, irinotecan and topotecan, are widely used in the treatment of colorectal, pancreatic, ovarian, and lung cancers [57,58,59,60,61]. This raises a number of questions that require further investigation: can DoG transcripts serve as markers of response to therapy with TOP1 inhibitors? Is it possible to suppress tumor growth by inhibiting the expression of specific DoG variants, or by targeting readthrough transcription in general, for instance, through factors that regulate it? Moreover, as mentioned above, some chimeric cis-SAGe transcripts have oncogenic properties, while others may serve as a source of neoantigens. Thus, the study of cis-SAGe transcripts may provide a basis for the development of antitumor therapies targeting such chimeric proteins.

For instance, the study [62] demonstrated that co-inhibition of TOP1 and bromodomain-containing protein 4 (BRD4) synergistically induces tumor regression. Comparative analysis of the nascent transcriptome alongside the recruitment of elongation and termination factors revealed that TOP1 and BRD4 co-inhibition disrupts the recruitment of transcription termination factors. As a result, RNA polymerases extend transcription far beyond normal gene termination sites, producing readthrough transcripts. When this occurs alongside replication, it interferes with replisome movement and causes DNA damage. Notably, the synergistic effect of TOP1 and BRD4 inhibition is relatively selective for cancer cells, mostly sparing normal cells and highlighting tumor susceptibility to transcriptional defects. This preclinical study provides insights into the therapeutic potential of combining TOP1 and BRD4 inhibitors for treating pancreatic carcinomas that depend on transcriptional and replicative oncogenic drivers.

The tissue- and stage-specific expression of DoG RNAs supports their potential as therapeutic targets and as biomarkers for prognosis and treatment in colorectal cancer [6]. Induction of DoG production may provide a therapeutic benefit, as evidenced by increased DoG levels in colorectal cancer cells following inhibition of TOP1 catalytic activity with camptothecin [63]. Consistent with this potential clinical benefit, TOP1 up-regulation, which is common in colorectal tumors, is associated with lower DoG numbers. Therefore, the therapeutic effect of camptothecin, a topoisomerase I inhibitor that stabilizes the TOP1-DNA cleavage complex and induces DNA damage, in tumors with elevated TOP1 may be partly mediated through enhanced DoG RNA production. However, further mechanistic studies are required to clarify how variations in TOP1 levels influence DoG RNA synthesis in human cancers. This suggests that multiple mechanisms likely underlie the influence of TOP1 on DoG production.

Another potential research direction involves the relationship between readthrough transcription and therapeutic response. For instance, suppression of the endonuclease CPSF73, a component of the cleavage and polyadenylation complex, leads to increased formation of readthrough transcripts. At the cellular level, degradation or inhibition of CPSF73 reduces migration, invasion, and self-renewal of triple-negative breast cancer cells, whereas *CPSF73* overexpression has the opposite effect. Consequently, CPSF73 is considered a promising novel target for anticancer therapy [14]. A similar scenario has been observed with inhibition of TOP1, which increases DoG production while its inhibitors simultaneously exhibit antitumor effects. It is worth investigating in more detail whether readthrough transcription can serve as a marker of therapeutic response, at least for certain classes of drugs, and whether readthrough transcription may contribute to the development of drug resistance.

These findings underscore the significance of DoGs and cis-SAGes within the transcript landscape, suggesting that they may have a greater role in disease and cancer biology than previously appreciated. Presented chimeric cis-SAGe transcripts that exhibit cancer type-specific expression demonstrate oncogenic properties and represent potential biomarkers or therapeutic targets.

## 5. DoG and Cis-SAGe Detection

**Figure 5:**
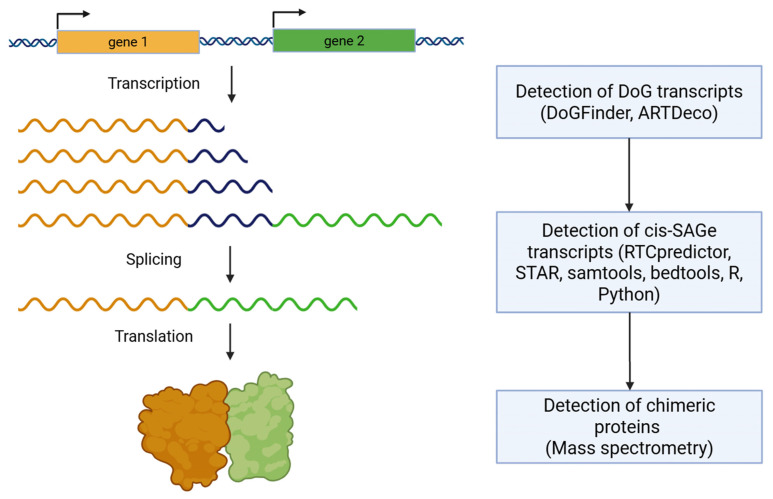
Pipeline for DoG and cis-SAGe detection. Specialized transcriptomic tools, such as ARTDeco and DoGFinder, are commonly used to identify DoGs. Based on RNA-seq data, cis-SAGe transcripts can be detected using ready-to-use tool RTCpredictor, from STAR outputs, through gene region annotation with samtools and bedtools, or with libraries in R and Python. In cases where a chimeric transcript is translated, mass spectrometry is employed to identify the resulting chimeric proteins.

As mentioned above, readthrough transcripts may act as molecular sponges for miRNAs [4,21], participate in maintaining chromatin structure [20], regulate gene expression as antisense RNAs [2], or repress the expression of genes through competitive mechanisms [22]. In particular, cis-SAGes are of notable clinical relevance. To date, numerous chimeric genes involved in tumorigenesis have been identified. Such chimeric genes represent important therapeutic targets in cancer, for example, *BCR::ABL*, *EML4::ALK*, and chimeric genes involving *NTRK1-3*, *RET*, *ROS1*, and others [64,65,66,67]. They can also serve as diagnostic and prognostic markers, such as *TMPRSS2::ERG*, *WWTR1::CAMTA1*, and chimeric genes involving *BRAF*, *ERBB2*, etc. [66,68,69]. These chimeric genes are typically formed as a result of chromosomal rearrangements. Such rearrangements are usually detected directly using fluorescence in situ hybridization (FISH) or indirectly by analyzing mRNA for the presence of fusion gene products [70,71,72]. The rapid development of RNA sequencing technology has enabled the discovery of a broad class of chimeric transcripts unrelated to genomic rearrangements but instead arising from alternative splicing. Typically, these chimeric transcripts are products of transcription from two or more co-oriented genes located in close proximity to each other. They originate when transcription of one gene fails to terminate at the expected site and instead continues into the intergenic region and into the neighboring gene. In routine RNA sequencing data analysis, such findings are often discarded as artifacts. However, recent studies demonstrate that at least some of these chimeric transcripts are functional. They can undergo translation, and the resulting chimeric proteins can be detected by Western blotting or mass spectrometry [3] (Figure 5). In addition to chimeric transcripts retaining an intact open reading frame, in many cases the fusion may disrupt the open reading frame, producing transcripts that function as non-coding RNAs. The same applies to readthrough transcripts that overlap an adjacent gene located in the opposite orientation, or that extend into intergenic regions. Thus, readthrough transcripts represent a large class of RNAs with either protein-coding or regulatory potential that remains poorly studied.

As a rule, readthrough transcripts remain outside the scope of standard RNA sequencing data analysis. Primarily, they are not taken into account because reads mapping to intergenic regions are not assigned to annotated genes. In cases where readthrough transcripts extend into an adjacent gene in the opposite orientation, they are filtered out as non-target reads. For co-oriented genes, readthrough reads are counted as target reads of the second gene. If, as a result of cis-splicing between these genes, a non-functional transcript is formed, such reads may distort the true expression profile of the second gene. In the case of cis-SAGe transcripts, when analyzing the output of chimeric gene detection software based on RNA sequencing data, they are typically discarded as artifacts. Therefore, investigation of the readthrough transcription phenomenon is necessary not only to gain more comprehensive insights into transcriptional regulation, but also to optimize RNA sequencing data analysis protocols [73].

To date, ready-to-use bioinformatics tools for the study of readthrough transcripts have begun to emerge [74,75,76,77,78,79,80] (Figure 5). For instance, ARTDeco [74] and DoGFinder [75] are designed for analyzing transcriptomic data obtained from short-read sequencing, while Readon is intended for long-read sequencing data [77]. Specialized tools for detecting cis-SAGe transcripts from RNA-seq data are also being developed, such as RTCpredictor [78]. We propose that the most reliable results are likely to be obtained using paired-end sequencing with longer read lengths. In cases where a chimeric transcript is translated, mass spectrometry is employed to identify the resulting chimeric proteins [3]. However, when analyzing transcriptomic data, it is essential to consider library preparation parameters (e.g., single-end versus paired-end sequencing, read number, and read length), as these factors can substantially affect the detection of readthrough transcription [73]. The advent of long-read sequencing technologies provides the opportunity to capture full DoG transcripts, thereby overcoming previous technical limitations [81]. This approach enables clarification of their splicing patterns and allows systematic assessment of whether DoG RNAs are preferentially enriched or depleted in specific RNA modifications [81,82].

Several cancer-related databases have been established to catalog identified chimeric transcripts; however, most are mainly dedicated to fusion transcripts resulting from intra- or inter-chromosomal rearrangements [83,84,85,86]. FusionGDB 2.0 (https://compbio.uth.edu/FusionGDB2/ (accessed on 9 September 2025)) [85] integrates 102,647 fusion genes derived from two major resources, ChiTaRS 5.0 [83] and ChimerDB 4.0 [86]. Collectively, these fusion events involve 26,688 genes corresponding to 17,300 UniProt accessions. The recently released ChiTaRS 8.0 features an expanded catalog of gene fusions with potential therapeutic relevance [87].

Although progress has been made in characterizing readthrough transcription in cancer, comprehensive large-scale comparisons between healthy and cancer tissues are still lacking. To address this, a reference dataset of readthrough transcripts in healthy human tissues was generated and integrated into the publicly accessible platform hhrtBase (http://www.hhrtbase.com/ (accessed on 9 September 2025)) [34]. The dataset includes 75,248 readthrough events originating from 11,692 genes across 43 healthy tissues, with all transcriptomic data derived from GTEx samples [88]. The analysis employed a standardized computational pipeline incorporating the STAR aligner [89] and ARTDeco [74]. Given that GTEx utilized non-stranded RNA-seq libraries, additional filtering was needed to exclude transcripts overlapping with genes encoded on the opposite strand.

Collectively, these resources provide a foundation for systematic exploration of the readthrough phenomenon and contribute to a deeper understanding of the prevalence and biological significance of readthrough transcription.

## 6. Conclusions

Recently, a new class of biomarkers—downstream-of-gene (DoG) transcripts, and their particular case, chimeric transcripts of neighboring genes—has attracted increasing attention. They represent extensions of gene transcripts with a length of 2 to 100 kbp and arise when RNA polymerase II fails to recognize transcription termination signals, causing the growing RNA to continue beyond the original gene and potentially extend into neighboring genes. Such transcripts can be synthesized both under normal conditions and in response to various stresses or pathological states. Notably, their formation is not a random process but depends on the type of tissue, donor age, and type of exposure. These transcripts can act as molecular sponges for miRNAs, participate in maintaining chromatin structure, influence the expression of neighboring genes through antisense regulation, and serve as a source of new protein products. Recent studies show that the formation of readthrough transcripts may be specific to tumor type and stage and may also serve as a prognostic marker of survival. A specific subset of DoG transcripts consists of chimeric splicing products derived from readthrough events that encompass two or more genes—cis-splicing between adjacent gene (cis-SAGe) transcripts. These, along with chimeric genes arising from chromosomal rearrangements, can be used as tumor markers and even as targets for anticancer therapy. Because chimeric cis-SAGe transcripts, and readthrough transcripts in general, are often filtered out during transcriptomic analyses, they represent a promising source of novel biomarkers and therapeutic targets. Studying readthrough transcription can also provide new insights into the mechanisms of gene expression regulation.

## Data Availability

No new data were created or analyzed in this study. Data sharing is not applicable to this article.

## References

[1] Chwalenia K., Qin F., Singh S., Tangtrongstittikul P., Li H. (2017). Connections between Transcription Downstream of Genes and Cis-SAGe Chimeric RNA. Genes.

[2] Vilborg A., Sabath N., Wiesel Y., Nathans J., Levy-Adam F., Yario T.A., Steitz J.A., Shalgi R. (2017). Comparative Analysis Reveals Genomic Features of Stress-Induced Transcriptional Readthrough. Proc. Natl. Acad. Sci. USA.

[3] Babiceanu M., Qin F., Xie Z., Jia Y., Lopez K., Janus N., Facemire L., Kumar S., Pang Y., Qi Y. (2016). Recurrent Chimeric Fusion RNAs in Non-Cancer Tissues and Cells. Nucleic Acids Res..

[4] Caldas P., Luz M., Baseggio S., Andrade R., Sobral D., Grosso A.R. (2024). Transcription Readthrough Is Prevalent in Healthy Human Tissues and Associated with Inherent Genomic Features. Commun. Biol..

[5] Bartz J., Jung H., Wasiluk K., Zhang L., Dong X. (2023). Progress in Discovering Transcriptional Noise in Aging. Int. J. Mol. Sci..

[6] Abe K., Maunze B., Lopez P.-A., Xu J., Muhammad N., Yang G.-Y., Katz D., Liu Y., Lauberth S.M. (2024). Downstream-of-Gene (DoG) Transcripts Contribute to an Imbalance in the Cancer Cell Transcriptome. Sci. Adv..

[7] Grosso A.R., Leite A.P., Carvalho S., Matos M.R., Martins F.B., Vítor A.C., Desterro J.M.P., Carmo-Fonseca M., de Almeida S.F. (2015). Pervasive Transcription Read-through Promotes Aberrant Expression of Oncogenes and RNA Chimeras in Renal Carcinoma. eLife.

[8] Sorokin M., Lyadov V., Suntsova M., Garipov M., Semenova A., Popova N., Guguchkin E., Heydarov R., Zolotovskaia M., Zhao X. (2025). Detection of Fusion Events by RNA Sequencing in FFPE versus Freshly Frozen Colorectal Cancer Tissue Samples. Front. Mol. Biosci..

[9] Buzdin A.A., Heydarov R.N., Golounina O.O., Suntsova M.V., Matrosova A.V., Bondarenko E.V., Roumiantsev S.A., Sorokin M.I., Kholodenko R.V., Kholodenko I.V. (2025). Transcriptome-Wide Analysis of Pituitary and Ectopic Adrenocorticotropic Hormone-Secreting Tumors. Cancers.

[10] Rosa-Mercado N.A., Zimmer J.T., Apostolidi M., Rinehart J., Simon M.D., Steitz J.A. (2021). Hyperosmotic Stress Alters the RNA Polymerase II Interactome and Induces Readthrough Transcription despite Widespread Transcriptional Repression. Mol. Cell.

[11] Dasilva L.F., Blumenthal E., Beckedorff F., Cingaram P.R., Gomes Dos Santos H., Edupuganti R.R., Zhang A., Dokaneheifard S., Aoi Y., Yue J. (2021). Integrator Enforces the Fidelity of Transcriptional Termination at Protein-Coding Genes. Sci. Adv..

[12] Vilborg A., Passarelli M.C., Yario T.A., Tycowski K.T., Steitz J.A. (2015). Widespread Inducible Transcription Downstream of Human Genes. Mol. Cell.

[13] Fianu I., Ochmann M., Walshe J.L., Dybkov O., Cruz J.N., Urlaub H., Cramer P. (2024). Structural Basis of Integrator-Dependent RNA Polymerase II Termination. Nature.

[14] Liu H., Heller-Trulli D., Moore C.L. (2022). Targeting the mRNA Endonuclease CPSF73 Inhibits Breast Cancer Cell Migration, Invasion, and Self-Renewal. iScience.

[15] Chwalenia K., Qin F., Singh S., Li H. (2019). A Cell-Based Splicing Reporter System to Identify Regulators of Cis-Splicing between Adjacent Genes. Nucleic Acids Res..

[16] Yao J., Ding D., Li X., Shen T., Fu H., Zhong H., Wei G., Ni T. (2020). Prevalent Intron Retention Fine-Tunes Gene Expression and Contributes to Cellular Senescence. Aging Cell.

[17] Qin F., Song Z., Babiceanu M., Song Y., Facemire L., Singh R., Adli M., Li H. (2015). Discovery of CTCF-Sensitive Cis-Spliced Fusion RNAs between Adjacent Genes in Human Prostate Cells. PLoS Genet..

[18] Zhang Y., Gong M., Yuan H., Park H.G., Frierson H.F., Li H. (2012). Chimeric Transcript Generated by Cis-Splicing of Adjacent Genes Regulates Prostate Cancer Cell Proliferation. Cancer Discov..

[19] Hennig T., Michalski M., Rutkowski A.J., Djakovic L., Whisnant A.W., Friedl M.-S., Jha B.A., Baptista M.A.P., L’Hernault A., Erhard F. (2018). HSV-1-Induced Disruption of Transcription Termination Resembles a Cellular Stress Response but Selectively Increases Chromatin Accessibility Downstream of Genes. PLoS Pathog..

[20] Heinz S., Texari L., Hayes M.G.B., Urbanowski M., Chang M.W., Givarkes N., Rialdi A., White K.M., Albrecht R.A., Pache L. (2018). Transcription Elongation Can Affect Genome 3D Structure. Cell.

[21] Alkan A.H., Akgül B. (2022). Endogenous miRNA Sponges. Methods in Molecular Biology.

[22] Hadar S., Meller A., Saida N., Shalgi R. (2022). Stress-Induced Transcriptional Readthrough into Neighboring Genes Is Linked to Intron Retention. iScience.

[23] Jia Y., Xie Z., Li H. (2016). Intergenically Spliced Chimeric RNAs in Cancer. Trends Cancer.

[24] Nacu S., Yuan W., Kan Z., Bhatt D., Rivers C.S., Stinson J., Peters B.A., Modrusan Z., Jung K., Seshagiri S. (2011). Deep RNA Sequencing Analysis of Readthrough Gene Fusions in Human Prostate Adenocarcinoma and Reference Samples. BMC Med. Genom..

[25] Lu G., Wu J., Zhao G., Wang Z., Chen W., Mu S. (2016). Abundant and Broad Expression of Transcription-Induced Chimeras and Protein Products in Mammalian Genomes. Biochem. Biophys. Res. Commun..

[26] Yuan C., Han Y., Zellmer L., Yang W., Guan Z., Yu W., Huang H., Liao D.J. (2017). It Is Imperative to Establish a Pellucid Definition of Chimeric RNA and to Clear Up a Lot of Confusion in the Relevant Research. Int. J. Mol. Sci..

[27] Vidal A.F. (2020). Read-through Circular RNAs Reveal the Plasticity of RNA Processing Mechanisms in Human Cells. RNA Biol..

[28] Vo J.N., Cieslik M., Zhang Y., Shukla S., Xiao L., Zhang Y., Wu Y.-M., Dhanasekaran S.M., Engelke C.G., Cao X. (2019). The Landscape of Circular RNA in Cancer. Cell.

[29] Chen Z., Huang L., Wang K., Zhang L., Zhong X., Yan Z., Liu B., Zhu P. (2022). rtcisE2F Promotes the Self-Renewal and Metastasis of Liver Tumor-Initiating Cells via N6-Methyladenosine-Dependent E2F3/E2F6 mRNA Stability. Sci. China Life Sci..

[30] Morgan M., Shiekhattar R., Shilatifard A., Lauberth S.M. (2022). It’s a DoG-Eat-DoG World-Altered Transcriptional Mechanisms Drive Downstream-of-Gene (DoG) Transcript Production. Mol. Cell.

[31] Alpert T., Straube K., Oesterreich F.C., Herzel L., Neugebauer K.M. (2020). Widespread Transcriptional Readthrough Caused by Nab2 Depletion Leads to Chimeric Transcripts with Retained Introns. Cell Rep..

[32] Herzel L., Straube K., Neugebauer K.M. (2018). Long-Read Sequencing of Nascent RNA Reveals Coupling among RNA Processing Events. Genome Res..

[33] Rutkowski A.J., Erhard F., L’Hernault A., Bonfert T., Schilhabel M., Crump C., Rosenstiel P., Efstathiou S., Zimmer R., Friedel C.C. (2015). Widespread Disruption of Host Transcription Termination in HSV-1 Infection. Nat. Commun..

[34] Mei Y., Cheng Z., Lu Y., Wu S., Chen X. (2025). Comprehensive Resource for Transcription Readthrough Events in Healthy Human Tissues. Sci. Data.

[35] Stoeger T., Grant R.A., McQuattie-Pimentel A.C., Anekalla K.R., Liu S.S., Tejedor-Navarro H., Singer B.D., Abdala-Valencia H., Schwake M., Tetreault M.-P. (2022). Aging Is Associated with a Systemic Length-Associated Transcriptome Imbalance. Nat. Aging.

[36] He H., Wang Y., Ye P., Yi D., Cheng Y., Tang H., Zhu Z., Wang X., Jin S. (2020). Long Noncoding RNA ZFPM2-AS1 Acts as a miRNA Sponge and Promotes Cell Invasion through Regulation of miR-139/GDF10 in Hepatocellular Carcinoma. J. Exp. Clin. Cancer Res..

[37] Rickman D.S., Pflueger D., Moss B., VanDoren V.E., Chen C.X., de la Taille A., Kuefer R., Tewari A.K., Setlur S.R., Demichelis F. (2009). SLC45A3-ELK4 Is a Novel and Frequent Erythroblast Transformation-Specific Fusion Transcript in Prostate Cancer. Cancer Res..

[38] Qin F., Song Z., Chang M., Song Y., Frierson H., Li H. (2016). Recurrent Cis-SAGe Chimeric RNA, D2HGDH-GAL3ST2, in Prostate Cancer. Cancer Lett..

[39] Tang Y., Qin F., Liu A., Li H. (2017). Recurrent Fusion RNA DUS4L-BCAP29 in Non-Cancer Human Tissues and Cells. Oncotarget.

[40] Varley K.E., Gertz J., Roberts B.S., Davis N.S., Bowling K.M., Kirby M.K., Nesmith A.S., Oliver P.G., Grizzle W.E., Forero A. (2014). Recurrent Read-through Fusion Transcripts in Breast Cancer. Breast Cancer Res. Treat..

[41] Wu P., Yang S., Singh S., Qin F., Kumar S., Wang L., Ma D., Li H. (2018). The Landscape and Implications of Chimeric RNAs in Cervical Cancer. eBioMedicine.

[42] Zhu D., Singh S., Chen X., Zheng Z., Huang J., Lin T., Li H. (2019). The Landscape of Chimeric RNAs in Bladder Urothelial Carcinoma. Int. J. Biochem. Cell Biol..

[43] Wu H., Singh S., Shi X., Xie Z., Lin E., Li X., Li H. (2019). Functional Heritage: The Evolution of Chimeric RNA into a Gene. RNA Biol..

[44] Wu H., Singh S., Xie Z., Li X., Li H. (2020). Landscape Characterization of Chimeric RNAs in Colorectal Cancer. Cancer Lett..

[45] Thomson T.M., Lozano J.J., Loukili N., Carrió R., Serras F., Cormand B., Valeri M., Díaz V.M., Abril J., Burset M. (2000). Fusion of the Human Gene for the Polyubiquitination Coeffector UEV1 with Kua, a Newly Identified Gene. Genome Res..

[46] Pflueger D., Mittmann C., Dehler S., Rubin M.A., Moch H., Schraml P. (2015). Functional Characterization of BC039389-GATM and KLK4-KRSP1 Chimeric Read-through Transcripts Which Are up-Regulated in Renal Cell Cancer. BMC Genom..

[47] Zhang L., Wang D., Han X., Guo X., Cao Y., Xia Y., Gao D. (2022). Novel Read-through Fusion Transcript Bcl2l2-Pabpn1 in Glioblastoma Cells. J. Cell. Mol. Med..

[48] Kumar-Sinha C., Kalyana-Sundaram S., Chinnaiyan A.M. (2012). SLC45A3-ELK4 Chimera in Prostate Cancer: Spotlight on Cis-Splicing. Cancer Discov..

[49] Qin F., Zhang Y., Liu J., Li H. (2017). SLC45A3-ELK4 Functions as a Long Non-Coding Chimeric RNA. Cancer Lett..

[50] Kang G., Yun H., Sun C.-H., Park I., Lee S., Kwon J., Do I., Hong M.E., Van Vrancken M., Lee J. (2016). Integrated Genomic Analyses Identify Frequent Gene Fusion Events and VHL Inactivation in Gastrointestinal Stromal Tumors. Oncotarget.

[51] Choi E.-S., Lee H., Lee C.-H., Goh S.-H. (2016). Overexpression of KLHL23 Protein from Read-through Transcription of PHOSPHO2-KLHL23 in Gastric Cancer Increases Cell Proliferation. FEBS Open Bio.

[52] Kolfschoten G.M., Pradet-Balade B., Hahne M., Medema J.P. (2003). TWE-PRIL; a Fusion Protein of TWEAK and APRIL. Biochem. Pharmacol..

[53] Li N., Zheng J., Li H., Deng J., Hu M., Wu H., Li W., Li F., Lan X., Lu J. (2014). Identification of Chimeric TSNAX-DISC1 Resulting from Intergenic Splicing in Endometrial Carcinoma through High-Throughput RNA Sequencing. Carcinogenesis.

[54] Gao S., Lin Z., Li C., Wang Y., Yang L., Zou B., Chen J., Li J., Feng D., Song Z. (2019). lncINS-IGF2 Promotes Cell Proliferation and Migration by Promoting G1/S Transition in Lung Cancer. Technol. Cancer Res. Treat..

[55] Cheng Y., Wang Y., Li J., Chang I., Wang C.-Y. (2017). A Novel Read-through Transcript JMJD7-PLA2G4B Regulates Head and Neck Squamous Cell Carcinoma Cell Proliferation and Survival. Oncotarget.

[56] Bond J., Tran Quang C., Hypolite G., Belhocine M., Bergon A., Cordonnier G., Ghysdael J., Macintyre E., Boissel N., Spicuglia S. (2018). Novel Intergenically Spliced Chimera, NFATC3-PLA2G15, Is Associated with Aggressive T-ALL Biology and Outcome. Mol. Cancer Res. MCR.

[57] Chai Y., Liu J.-L., Zhang S., Li N., Xu D.-Q., Liu W.-J., Fu R.-J., Tang Y.-P. (2024). The Effective Combination Therapies with Irinotecan for Colorectal Cancer. Front. Pharmacol..

[58] Gupta A., De Jesus-Acosta A., Zheng L., Lee V., Kamel I., Le D., Pishvaian M., Laheru D. (2023). Clinical Outcomes of Liposomal Irinotecan in Advanced Pancreatic Adenocarcinoma Patients Previously Treated with Conventional Irinotecan-Based Chemotherapy: A Real-World Study. Front. Oncol..

[59] Tate S., Nishikimi K., Matsuoka A., Otsuka S., Kato K., Takahashi Y., Shozu M. (2021). Tailored-Dose Chemotherapy with Gemcitabine and Irinotecan in Patients with Platinum-Refractory/Resistant Ovarian or Primary Peritoneal Cancer: A Phase II Trial. J. Gynecol. Oncol..

[60] Seto Z., Takata N., Murayama N., Tokui K., Okazawa S., Kambara K., Imanishi S., Miwa T., Hayashi R., Matsui S. (2021). Irinotecan Monotherapy as Third- or Further-Line Treatment for Patients with Small Cell Lung Cancer. Tumori.

[61] Reyhanoglu G., Smith T. (2025). Irinotecan. StatPearls.

[62] Cameron D.P., Grosser J., Ladigan S., Kuzin V., Iliopoulou E., Wiegard A., Benredjem H., Jackson K., Liffers S.T., Lueong S. (2023). Coinhibition of Topoisomerase 1 and BRD4-Mediated Pause Release Selectively Kills Pancreatic Cancer via Readthrough Transcription. Sci. Adv..

[63] Khobta A., Ferri F., Lotito L., Montecucco A., Rossi R., Capranico G. (2006). Early Effects of Topoisomerase I Inhibition on RNA Polymerase II along Transcribed Genes in Human Cells. J. Mol. Biol..

[64] Cilloni D., Saglio G. (2012). Molecular Pathways: BCR-ABL. Clin. Cancer Res..

[65] Desai A.V., Robinson G.W., Gauvain K., Basu E.M., Macy M.E., Maese L., Whipple N.S., Sabnis A.J., Foster J.H., Shusterman S. (2022). Entrectinib in Children and Young Adults with Solid or Primary CNS Tumors Harboring *NTRK*, *ROS1*, or *ALK* Aberrations (STARTRK-NG). Neuro-Oncology.

[66] Sorokin M., Rabushko E., Rozenberg J.M., Mohammad T., Seryakov A., Sekacheva M., Buzdin A. (2022). Clinically Relevant Fusion Oncogenes: Detection and Practical Implications. Ther. Adv. Med. Oncol..

[67] Zakharova G., Suntsova M., Rabushko E., Mohammad T., Drobyshev A., Seryakov A., Poddubskaya E., Moisseev A., Smirnova A., Sorokin M. (2024). A New Approach of Detecting ALK Fusion Oncogenes by RNA Sequencing Exon Coverage Analysis. Cancers.

[68] Marín-Aguilera M., Reig Ò., Milà-Guasch M., Font A., Domènech M., Rodríguez-Vida A., Carles J., Suárez C., Del Alba A.G., Jiménez N. (2019). The Influence of Treatment Sequence in the Prognostic Value of TMPRSS2-ERG as Biomarker of Taxane Resistance in Castration-Resistant Prostate Cancer. Int. J. Cancer.

[69] Errani C., Zhang L., Shao S.Y., Hajdu M., Singer S., Maki R.G., Healey J.H., Antonescu C.R. (2011). A Novel WWTR1-CAMTA1 Gene Fusion Is a Consistent Abnormality in Epithelioid Hemangioendothelioma of Different Anatomic Sites. Genes. Chromosomes Cancer.

[70] Rabushko E., Sorokin M., Suntsova M., Seryakov A.P., Kuzmin D.V., Poddubskaya E., Buzdin A.A. (2022). Experimentally Deduced Criteria for Detection of Clinically Relevant Fusion 3′ Oncogenes from FFPE Bulk RNA Sequencing Data. Biomedicines.

[71] Cruz-Rico G., Avilés-Salas A., Segura-González M., Espinosa-García A.M., Ramírez-Tirado L.A., Morales-Oyarvide V., Rojas-Marín C., Cardona A.-F., Arrieta O. (2017). Diagnosis of EML4-ALK Translocation with FISH, Immunohistochemistry, and Real-Time Polymerase Chain Reaction in Patients with Non-Small Cell Lung Cancer. Am. J. Clin. Oncol..

[72] Ali J., Khan S.A., Rauf S.-E.-, Ayyub M., Ali N., Afridi N.K. (2017). Comparative Analysis of Fluorescence In Situ Hybridization and Real Time Polymerase Chain Reaction in Diagnosis of Chronic Myeloid Leukemia. J. Coll. Physicians Surg. Pak. JCPSP.

[73] Singh S., Li H. (2021). Comparative Study of Bioinformatic Tools for the Identification of Chimeric RNAs from RNA Sequencing. RNA Biol..

[74] Roth S.J., Heinz S., Benner C. (2020). ARTDeco: Automatic Readthrough Transcription Detection. BMC Bioinform..

[75] Wiesel Y., Sabath N., Shalgi R. (2018). DoGFinder: A Software for the Discovery and Quantification of Readthrough Transcripts from RNA-Seq. BMC Genom..

[76] Melnick M., Gonzales P., Cabral J., Allen M.A., Dowell R.D., Link C.D. (2019). Heat Shock in *C. Elegans* Induces Downstream of Gene Transcription and Accumulation of Double-Stranded RNA. PLoS ONE.

[77] Chen S., Wang H., Zhang D., Chen R., Luo J. (2024). Readon: A Novel Algorithm to Identify Read-through Transcripts with Long-Read Sequencing Data. Bioinformatics.

[78] Singh S., Shi X., Haddox S., Elfman J., Ahmad S.B., Lynch S., Manley T., Piczak C., Phung C., Sun Y. (2024). RTCpredictor: Identification of Read-through Chimeric RNAs from RNA Sequencing Data. Brief. Bioinform..

[79] Modestov A., Buzdin A., Prassolov V. (2025). Omics Wise Analysis of RNA Splicing. Handbook of Translational Transcriptomics.

[80] Musatov I., Sorokin M., Buzdin A. (2025). Detection of Fusion Transcripts by RNA-Sequencing Data. Handbook of Translational Transcriptomics.

[81] Logsdon G.A., Vollger M.R., Eichler E.E. (2020). Long-Read Human Genome Sequencing and Its Applications. Nat. Rev. Genet..

[82] Modestov A., Buzdin A., Suntsova M. (2025). Unveiling RNA Editing by ADAR and APOBEC Protein Gene Families. Front. Biosci.-Landmark.

[83] Balamurali D., Gorohovski A., Detroja R., Palande V., Raviv-Shay D., Frenkel-Morgenstern M. (2020). ChiTaRS 5.0: The Comprehensive Database of Chimeric Transcripts Matched with Druggable Fusions and 3D Chromatin Maps. Nucleic Acids Res..

[84] Barresi V., Cosentini I., Scuderi C., Napoli S., Di Bella V., Spampinato G., Condorelli D.F. (2019). Fusion Transcripts of Adjacent Genes: New Insights into the World of Human Complex Transcripts in Cancer. Int. J. Mol. Sci..

[85] Kim P., Tan H., Liu J., Lee H., Jung H., Kumar H., Zhou X. (2022). FusionGDB 2.0: Fusion Gene Annotation Updates Aided by Deep Learning. Nucleic Acids Res..

[86] Jang Y.E., Jang I., Kim S., Cho S., Kim D., Kim K., Kim J., Hwang J., Kim S., Kim J. (2020). ChimerDB 4.0: An Updated and Expanded Database of Fusion Genes. Nucleic Acids Res..

[87] DSouza D., Bik L., Giwa O., Cohen S., Barazany H.L., Siegal T., Frenkel-Morgenstern M. (2024). ChiTaRS 8.0: The Comprehensive Database of Chimeric Transcripts and RNA-Seq Data with Applications in Liquid Biopsy. Nucleic Acids Res..

[88] Lonsdale J., Thomas J., Salvatore M., Phillips R., Lo E., Shad S., Hasz B., Walters G., Garcia F., Young N. (2013). The Genotype-Tissue Expression (GTEx) Project. Nat. Genet..

[89] Dobin A., Davis C.A., Schlesinger F., Drenkow J., Zaleski C., Jha S., Batut P., Chaisson M., Gingeras T.R. (2013). STAR: Ultrafast Universal RNA-Seq Aligner. Bioinformatics.

